# Are medical associations’ paid parental leave recommendations instituted for United States medical school faculty?

**DOI:** 10.1080/10872981.2025.2487656

**Published:** 2025-04-02

**Authors:** Hannah Gurley, Rebecca S. Lufler, Brian J. Goldberg, Christopher Ferrigno, Adam B. Wilson

**Affiliations:** a Tufts University School of Medicine, Boston, MA, USA; b Department of Medical Education, Tufts University School of Medicine, Boston, MA, USA; c Rush Medical College, Rush University, Chicago, IL, USA; d Department of Anatomy and Cell Biology, Rush University, Chicago, IL, USA

**Keywords:** Parental leave, faculty benefits, leave policies, medical schools, academic medicine, maternity leave, paternity leave

## Abstract

Longer paid parental leaves have many well-documented biopsychosocial benefits for parents and children. However, many United States (U.S.) employers do not offer 8–12 weeks of paid parental leave as recommended by medical associations such as The American College of Obstetricians and Gynecologists, the American Academy of Pediatrics, and the American Medical Association. This study compared and summarized the quality of parental leave policies offered across U.S. allopathic medical schools to determine their alignment with medical associations’ recommendations. Parental leave policies were analyzed to determine 1) whether employers offered standalone parental leave policies and/or relied on the Family Medical Leave Act, 2) who received parental leave, 3) whether leave was paid or unpaid, and 4) the number of paid weeks offered, if any. Differences in leave durations were compared according to Carnegie classifications, school control, and geographic region. Of the 134 (85.9%; 134/156) allopathic medical schools with retrievable policies, one-fifth (21.6%; 29/134) offered 12 weeks or more of fully compensated birthing parent leave. Schools offered an average of 6.72 weeks (median = 6) of paid birthing parent leave and 5.82 weeks (median = 6) of paid non-birthing parent leave. Private (*p* < 0.001) and Northeast (*p* < 0.001) schools offered more weeks of paid birthing parent leave. Despite the benefits of longer paid parental leaves, over three-quarters of parental leave policies used by allopathic medical schools did not offer faculty 12 weeks of fully paid birthing parent (78.4%; 105/134) or non-birthing parent leave (84.3%; 113/134). This suggests that most parental leave policies offered to academic medicine faculty are misaligned with medical associations’ recommendations.

## Introduction

According to data from the Organisation for Economic Co-operation and Development and World Bank, the United States (U.S.) ranks last in a list of countries with a high Human Development Index for childcare and paid parental leave [1,2]. Almost all industrialized countries have established statutory minimums for paid leave following childbirth, except for the United States [3]. Lower quality parental leave benefits are considered commonplace within the healthcare sector and among U.S. medical schools, both of which fail to rival the employer benefits of other industries [4]. According to one analysis, the healthcare industry ranks last for the average number of weeks of paid parental leave, at only 4 weeks for primary and secondary caregivers [5]. While medical institutions commonly adopt guidelines from medical associations for clinical decision-making practices, evidence of their reliance on such guidelines is lacking when considering policy implications for faculty parental leave benefits [6]. Like many other U.S. employers, medical schools are said to provide parental leave benefits that are misaligned with current medical associations’ recommendations [6,7]. This work conducts a large-scale analysis of parental leave policies offered to medical school faculty to help monitor progress toward alignment.

The American Academy of Pediatrics (AAP) and the American Medical Association (AMA) both endorse 12 weeks of paid parental leave to improve birthing parents’ mental health and overall child well-being (i.e., attendance at pediatrician visits, timely immunizations, and improved infant-caregiver attachment) [8,9]. A 2023 statement from the American College of Obstetricians and Gynecologists (ACOG) endorses at least 8 weeks of fully paid parental leave as essential, citing health benefits such as decreased infant mortality, improved health outcomes for the birthing parent and newborn, and longer-term retention of females in the workforce [10]. Despite these clear recommendations and their benefits, history would suggest that enacting ubiquitous and substantive changes to parental leave benefits may be more of a distant aspiration than an immediate and tangible reformation. For example, over 25 years ago, four gastroenterology societies called for a minimum of 8 weeks of parental leave for gastroenterologists [11]. Since this call to action, very few structural or meaningful changes have been realized [11].

### Benefits of paid parental leave

Paid parental leave is critical for appropriate perinatal care and improves the well-being of the birthing parent and child [12]. Extensive research supports longer paid parental leaves and suggests that postpartum care should be an ‘ongoing process’ of up to 12 weeks to reap positive health benefits [13–17]. Longer paid parental leave for birthing parents is associated with increased rates of breastfeeding and immunizations, decreased infant mortality, and a lower incidence of postpartum depression [18–20]. Non-birthing parental leave improves engagement and bonding, promoting child health and development (i.e., higher cognitive test scores) [21].

### Study objective

This large-scale document analysis represents the most current and comprehensive summary of parental leave policies used by U.S. allopathic medical schools. This work investigates the proportion of parental leave policies that adhere to current medical associations’ recommendations and evaluates whether strides toward equality between birthing and non-birthing parent leave policies have been made. Two related studies only examined parental leave policies in medical schools ranked by the US News & World Report (*n* = 90) or schools on the top-10 lists for both funding by the National Institutes of Health and academic ranking by the US News & World Report (*n* = 12), thereby limiting their scope [22,23]. The present study incorporates the Family Medical Leave Act (FMLA) and state-based paid family leave policies into a holistic assessment of leave offerings made available to parents in academic medicine.

## Materials and methods

This document analysis was not subject to ethics review as it did not involve research on human subjects.

### Study protocol

In 2022, employment benefits data were compiled across U.S. allopathic medical schools by searching institutions’ publicly available parental leave policies via their institutional and human resources websites. After pilot testing, an equivalent benefits analysis of the 43 osteopathic medical schools was deemed unfeasible given the limited number of publicly available benefits documents. Hence, this study explored only parental leave policies related to U.S. allopathic medical schools.

Parental leave policies were isolated from a larger benefits dataset (including time-off, retirement benefits, etc.) and separately evaluated and summarized, given their unique richness and features. Parental leave benefits were evaluated only for full-time basic science and clinical faculty fully contracted through the medical school and/or university offering the benefit. This study did not account for employer benefits offered to clinical faculty contracted through independent medical groups (i.e., separate corporations distinct from the medical school/university). Human resources representatives were contacted to verify and clarify the accuracy of ambiguous and/or undocumented benefits. Two authors (RL and HG) independently extracted parental leave data and compared outcomes to determine consistency. All interpretational discrepancies were resolved through consensus to yield the final dataset.

Table 1 provides an overview of categories, variables, and definitions included in the parental leave policy analysis. Parental leave policies were analyzed to determine: a) whether employers offered standalone parental leave policies and/or relied on the Family Medical Leave Act, b) the recipient of the benefit, c) whether leave was paid or unpaid, d) the number of paid weeks offered, if any, e) policy name terminology, and f) method(s) of family building (e.g., birth, adoption, fostering).

**Table 1.** Reference table for policy analysis.

Category	Variables	Definitions	Notes
Family Medical Leave Act (FMLA)	FMLA with No Separate Policy	Medical schools that referenced FMLA but had no other parental leave policy documented in their benefits packages	If a school used the only FMLA as their parental leave policy, 12 weeks of unpaid leave was recorded for both parents.
	FMILA with a Separate Policy	Medical schools with both a defined parental leave policy and FMLA in their benefits packages	If a school used the FMLA as their parental leave policy AND the school was in one of the states with laws requiring paid parental leave (in effect since 2022 or earlier), the amount of paid leave required by the state was recorded for both parents if no other data were available.
	FMLA with Stipulations	Medical schools with certain requirements surrounding the usage of FMLA for parental leave (e.g., stipulating an employee must exhaust vacation days, sick days, or paid time off (PTO) during their FMLA leave)	
	FMLA with Options	Medical schools that allowed, but did not require, stipulations surrounding the usage of FMLA for parental leave (e.g., employees may use PTO or sick time for pay during FMLA leave, but this is not required)	
Parental Leave Eligibility	Eligible Parents	Medical schools that offered leave to eligible parents.	Schools that used the FMLA as their parental leave policy or lacked an explicit parental leave policy were combined since both parents received the same paucity of parental leave benefits beyond what is required by federal law.
Compensation	Paid Eligible Parent Leave	Parental leave with any amount of compensation.	Schools using only FMLA as their parental leave policy were documented as ‘paid’ if the school was in a state with a Paid Family Leave law (in effect since 2022 or earlier) and ‘unpaid’ if in any other state.
	Unpaid Eligible Parental Leave	Parental leave with no compensation.	
Method of Family Building	Birth	Medical schools that offered parental leave for the birth of a child.	Schools using only FMLA as their parental leave policy were evaluated based on FMLA eligibility criteria, which declares no difference between birthing, adoption, or foster placement.
	Adoption	Medical schools that offered parental leave for the adoption of a child.	
	Fostering	Medical schools that offered parental leave for the foster placement of a child.	

### Family medical leave act (FMLA) and states with required paid family leave

The FMLA is a federal law enacted in 1993 that provides up to 12 weeks of unpaid job-protected leave for American workers to care for themselves and/or family members when they experience a qualifying event [24]. According to the U.S. Department of Labor, birth, adoption, or foster placement of a child are considered qualifying events. To utilize FMLA, employees must have worked for their employer for at least 1,250 hours over the preceding 12 months, and the employer must have at least 50 employees. When a school’s parental leave policy fell under the FMLA, additional categories were evaluated (e.g., stipulations or options as defined in Table 1).

When this manuscript was written, nine states and the District of Columbia had laws requiring paid family leave (Table 2) [25–35]. An additional four states are enacting similar laws in 2026, and two others provide voluntary leave options through private insurance markets (Table 2) [36–41]. The details of these benefits vary by state. Most states provide 12 weeks of parental leave with at least some proportion of salary compensation. The two exceptions include Rhode Island offering 6 weeks and California offering up to 8 weeks of paid family leave at a 60–70% wage replacement rate [42].

**Table 2.** States with paid family leave laws.

States with existing Paid Family Leave Laws	States with approved PaidFamily Leave Laws to be enacted in 2026	States with Voluntary PaidFamily Leave Systems (through private insurance)
California Colorado Connecticut District of Columbia Massachusetts New Jersey New York Oregon Rhode Island Washington	Delaware Maine Maryland Minnesota	New Hampshire* Vermont

*Enrollment begins December 1, 2024.

### School characteristics

Schools were categorized according to their Carnegie classifications, school control (i.e., public versus private), and Association of American Medical College’s (AAMC) regional designation to determine whether school features explain differences in parental leave policies. Carnegie classification groups included: R1-very high research activity, R2-high research activity, Special focus four-year: research institutions, and Special focus four-year: medical schools & centers. For school control, private for-profit and private not-for-profit schools were combined as private schools. The four AAMC regions included the Central, Northeast, Southern, and Western regions.

### Statistical analysis

Data were exported to Microsoft Excel (Version 2306, Build 16.0.16625.42305) for organization and cleaning and were analyzed using SAS version 9.4 (Cary, North Carolina) and SPSS version 26 (Armonk, New York). Outcomes are reported as descriptive statistics. Pearson’s Chi-square analysis tested differences in the proportion of schools with or without retrievable policies by AAMC region, school control, and Carnegie classification. Differences in paid parental leave durations between schools’ Carnegie classifications, school control, and AAMC regional designations were compared using a one-way ANOVA. Effect sizes were measured using partial eta-squared and interpreted as either a small (η [2] = 0.01–0.05), medium (η [2] = 0.06–0.13), or large effect (η [2]≥0.14) [43].

## Results

Of the 156 total U.S. allopathic medical schools queried in this study, 134 (85.9%) had retrievable parental leave policies. Polices were collected from allopathic medical schools across all four AAMC regions (Central = 23.1%, 31/134; Northeast = 26.9%, 36/134; Southern = 34.3%, 46/134; Western = 15.7%, 21/134). Systemic bias by region was unlikely as a Chi-square analysis detected no statistically significant differences (*p* = 0.880) in the proportion of schools with or without retrievable policies by region. While the proportion of public schools with retrievable policies (67.9%, 91/134) was significantly higher (*p* = 0.001) than that of private schools (32.1%, 43/134), this was expected due to the higher proportion of public allopathic medical schools (62.6%, 97/155) in existence. Parental leave policies were also retrieved from a higher proportion (*p* < 0.001) of R1 institutions (60.6%, 80/132) than any other classification (≤18.2%, ≤24/132) because more than half of medical schools (53.3%, 80/150) are affiliated with R1 institutions.

### Paid versus unpaid parental leave

On average, the 134 parental leave policies offered 6.72 weeks (median = 6) of paid birthing parent leave and 5.82 weeks (median = 6) of paid non-birthing leave. Forty-two (31.3%; 42/134) parental leave policies offered at least 12 weeks of birthing leave with some amount of pay. Only 29 policies (21.6%; 29/134) offered 12 weeks of birthing parent leave with full compensation. One-quarter (26.1%; 35/134) of the policies offered at least 12 weeks of non-birthing parent leave with some amount of pay, while 15.7% (21/134) offered 12 weeks of paid non-birthing parent leave with full compensation.

In addition to offering paid parental leave, several schools also offered unpaid birthing parent leave (32.1%; 43 of 134) and unpaid non-birthing parent leave (29.1%; 39 of 134), intended for use after exhausting paid leave offerings. Table 3 summarizes the amount of parental leave offered. Table 4 summarizes the amount of additional unpaid parental leave at schools offering paid parental leave.

**Table 3.** Proportion of U.S. Allopathic medical schools offering parental leave according to leave durations in weeks.

Allotment in Weeks	Number of Schools (% of 134)			
	Partially or Fully Paid Birthing Parent Leave*	Unpaid Birthing Parent Leave Only	Partially or Fully Paid Non-birthing Parent Leave*	Unpaid Non-birthing Parent Leave Only
0 weeks (i.e., No explicit parental leave, must use sick/vacation days; FMLA only)	–	–	–	4 (3.0%)
1–4 weeks	10 (7.5%)	0	16 (11.9%)	0
5–6 weeks	24 (17.9%)	1 (0.75%)	24 (17.9%)	1 (0.75%)
7–8 weeks	18 (13.4%)	0	16 (11.9%)	0
9–11 weeks	3 (2.2%)	0	1 (0.75%)	1 (0.75%)
≥12 weeks	42 (31.3%)	36 (26.9%)	35 (26.1%)	36 (26.9%)
SUBTOTAL	97 (72.4%)	37 (27.6%)	92 (68.7%)	42 (31.3%)
TOTAL	134 (100%)		134 (100%)	
Summary: Proportion of schools misaligned with AMA and AAP recommendations by offering <12 weeks of fully paid parental leave.	78.4% (105/134)		84.3% (113/134)	

*Paid leave is defined as leave with any amount of pay, either full or partial.

**Table 4.** Weeks of additional unpaid parental leave at U.S. allopathic medical schools already offering some paid parental leave.

Allotment in Weeks	Number (%) of Schools	
	Additional Unpaid Birthing Parent Leave	Additional Unpaid Non-birthing Parent Leave
1–4 weeks	4 (3.0%)	2 (1.5%)
5–6 weeks	8 (23.5%)	9 (6.7%)
7–8 weeks	5 (3.7%)	5 (3.7%)
9–12 weeks	3 (2.2%)	1 (0.75%)
≥12 weeks	23 (17.2%)	22 (16.4%)
TOTAL	97 (72.4%)	91 (67.9%)

### Parental leave via FMLA or paid family leave laws

At 59.0% of medical schools (79/134), the parental leave policies explicitly fell under FMLA, meaning the available information detailed how FMLA was incorporated into the leave policies. A smaller subset of schools offered FMLA as their only parental leave option (18.7%; 25/134), while others offered a separate parental leave policy that was in addition to or ran concurrently with an FMLA policy (40.3%; 54/134).

Although FMLA is federally protected for all qualifying workers, employers can create stipulations or options surrounding this leave time. Eight medical schools (6.0%; 8/134) specified stipulations for FMLA leave requiring faculty to exhaust paid time off (PTO) or sick pay during their leave. An additional 45 of 134 schools (33.6%) allowed options for exhausting accrued PTO for pay but did not require it.

While six states and the District of Columbia require 12 weeks of at least partially paid leave through their various paid family leave laws, these states and their associated medical schools represent a minority. Currently, 43 of the 134 medical schools (32.33%) are located in states with state-based paid family leave laws, with benefits payments often around 60–70% of an employee’s income. California, home to 16 allopathic medical schools, requires 8 weeks of parental leave through its paid family leave law with partial wage recovery. In Rhode Island, home to one allopathic medical school, eligible claimants may receive up to 6 weeks of paid parental leave through the Temporary Caregiver Insurance law.

### Policy names

The language surrounding parental leave policies varied, with the most common policy names being ‘parental leave’ (59 schools, 44.36%), ‘family leave’ (23 schools, 17.29%), and ‘maternity leave’ (12 schools, 9.02%). These terms and other nomenclature are summarized in Table 5.

**Table 5.** Parental leave policy terminology.

Designation	Number of Schools
Parental Leave	59 (44.03%)
Family Leave	23 (17.16%)
Maternity Leave	12 (8.96%)
Childbearing Leave	6 (4.48%)
Childrearing Leave	3 (2.24%)
Primary Caregiver Leave	3 (2.24%)
Childbirth Leave	2 (1.50%)
Child Bonding Leave	2 (1.50%)
Child Care Leave	2 (1.50%)
Baby Bonding Time	1 (0.75%)
Care Giving Leave	1 (0.75%)
Parental Workload Relief Plan	1 (0.75%)

### Birthing vs. adoption vs. fostering

Based on the documented language, 87 parental leave policies (64.9%) explicitly stated there was no difference in parental leave for the birth of a biological child versus the adoption or fostering of a child. For policies with specified differences in benefits (*n* = 26), the most common difference was additional time off for the birthing parent (*n* = 17).

### Comparisons of paid birthing parent leave by school characteristics

**Figure 1.**
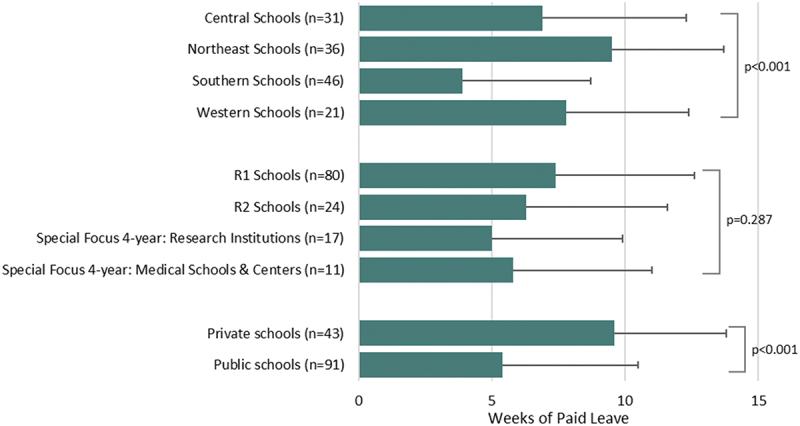
Mean weeks of paid birthing parent leave by school characteristics.The four Carnegie classifications sum to 132 instead of 134. One excluded school was classified as ‘Doctoral/Professional’. The second excluded school was not discoverable in the Carnegie database.

Private schools offered more weeks of paid birthing leave than public schools (*p* < 0.001; η [2] = 0.141 (large effect); Figure 1). On average, Northeast medical schools offered 9.5 weeks of paid birthing parent leave compared to 3.9 weeks offered by Southern schools (*p* < 0.001; η [2] = 0.182 (large effect); Figure 1). No significant differences were identified for the number of weeks of paid birthing parent leave according to Carnegie classification groups (*p* = 0.287).

## Discussion

This study summarized the current state of faculty parental leave policies across U.S. allopathic medical schools and determined the proportion of parental leave policies that adhere to current medical associations’ recommendations. Over 75% of allopathic medical schools did not offer faculty 12 weeks of a fully paid birthing parent or non-birthing parent leave and, thus, did not align with current medical associations’ recommendations. Nearly 20% of schools utilized FMLA as their sole parental leave policy, allowing only unpaid leave for eligible faculty. Sixty-five percent of policies offered equal amounts of leave across all family building methods (i.e., natural, adoption, fostering, or surrogacy). Private and Northeast schools offered more weeks of paid birthing parent leave.

### Majority of parental leave policies offered to medical school faculty do not align with medical associations’ recommendations

Three U.S. medical associations endorse paid parental leave ranging from 8–12 weeks (i.e., ACOG: 8 weeks; AMA and AAP: 12 weeks) due to its numerous benefits for children, mothers, and families [9,10,44,45]. Despite this guidance, 41% (55/97) of parental leave policies used by medical schools offered birthing parents less than 12 weeks of leave with full or partial pay (Table 3), and an additional 28% (37/134) offered leave without pay. On average, parental leave policies offered to medical school faculty (i.e., 6.72 weeks for birthing parents and 5.82 weeks for non-birthing parents) accounted for only half of the 12 weeks endorsed by the American Medical Association and the American Academy of Pediatrics. Only one-fifth of schools (21.6%; 29 of 134) provided 12 weeks of birthing parent leave with full compensation. According to the American Psychological Association, in the absence of a paid parental leave, faculty at these medical schools are more likely to cut their parental leave short, experience more depressive symptoms, discontinue breastfeeding, and face financial hardships after the arrival of their child [44].

### The utilization of FMLA by U.S. Medical schools

While state-based paid family leave establishes some required minimums, 25 (18.66%) medical schools solely managed faculty parental leave through federal law under the FMLA; a strictly unpaid time-off entitlement. With no supplemental parental leave benefit, schools that rely on the FMLA alone offer 12 weeks of unpaid leave, provided an employee has worked at the medical school for at least one year (or 1,250 hours) before the start of the parental leave. A 2018 study of the general public found that paid parental leave is associated with a 47% decrease in infant re-hospitalizations and a 51% decrease in re-hospitalization for the birthing parent in the year following childbirth compared to those who take unpaid leave or no leave [46]. Positive paid leave outcomes are thought to be due to prompt access to care, on-time child vaccinations, and improved mental health for the birthing parent [46,47].

In addition to not providing paid parental leave, eight medical schools had FMLA stipulations requiring the exhaustion of all accrued vacation, PTO, and sick time concurrent with their FMLA leave. This stipulation could leave new parents with no accrued time off, resulting in fewer options and little flexibility for handling personal and/or child illnesses, appointments, and other personal or family needs upon returning to work. By using state and federal government documents in place of explicit parental leave policies, some institutions may be missing an opportunity to create school-specific benefits that help to promote a local culture of wellness and recognition that faculty health is just as important as patient health.

### Parental leave disparities

Parity in parental leave between female and male faculty has significant implications for workforce equity and the well-being of the birthing parent and child [48]. Within academic medicine, inadequate parental leave benefits have been associated with greater career dissatisfaction, higher rates of burnout, and lower retention of female faculty [49,50]. Females often assume more domestic and childcare responsibilities than their male partners, even when their career obligations are equal or greater [51–53]. Parity in parental leave between birthing and non-birthing parents increases males’ contributions to childcare responsibilities and provides equal access to benefits for those starting a family through adoption, fostering, or surrogacy [54,55].

Providing equal and paid parental leave may also be integral in helping to close the pay gap between males and females. While the number of female faculty at U.S. medical schools has increased over the past 20 years, retention, advancement, and compensation among female faculty have consistently remained below that of their male counterparts [56,57]. Of the many possible explanations for these disparities, one significant contributing factor is that females are more likely to take a leave of absence for child care [58]. Ensuring that paid parental leave for both non-birthing and birthing parents is adequate, equitable, and protected from penalizations may help to alleviate some of the disparities frequently experienced by the birthing parent [14]. Research shows that up to 86% of non-birthing parents limit or choose not to take parental leave when offered only unpaid leave. Conversely, parental leave is often necessary for the birthing parent to provide adequate time for physical healing [59]. This creates a scenario whereby predominately female faculty may have no choice but to take unpaid leave after the birth of a child and, therefore, a possible reduction in income, while male faculty continue working to avoid lost wages. Subsequently, by remaining at work, male faculty may be rewarded with increased opportunities and responsibilities, ultimately contributing to higher ratios of males in leadership positions [50]. Longer paid non-birthing parent leave has been shown to increase male contributions to childcare and household chores during the leave and beyond while simultaneously having a ‘positive effect on female labor force participation and wages’ [21]. While 96.3% of medical school employers provided some amount of parental leave to both birthing and non-birthing parents, only one-third (31.3%; Table 3) of employers offered unpaid non-birthing parent leave, and 3.0% (4/134, Table 3) did not offer a clear or accessible non-birthing parent leave policy, aside from FMLA.

### The importance of inclusivity irrespective of family building method

The majority (65%; 87/34) of parental leave policies explicitly stated there was no difference in the amount of leave offered based on the method of family building. However, this outcome is mainly due to the schools that utilized FMLA for their policies. This has important implications for those starting families through adoption, fostering, or surrogacy. This consideration is particularly important for female physicians, who face infertility rates as high as 25%, double that of the general population [60]. On average, female physicians delay childbearing by 7 years [61]. This, along with sleep deprivation, poor diet, limited opportunities to exercise, and increased stress, have been theorized to contribute to an increased incidence of infertility among female physicians and a greater risk of pregnancy complications [62]. Given these significant pregnancy barriers/challenges for female medical professionals, parental leave policies that explicitly recognize alternative methods of family building are increasingly important. Most policies utilized inclusive language to describe parental leaves by using gender-neutral phrases such as ‘parental leave’ (44.4%; 59/134) or ‘family leave’ (17. 9%; 23/134), while only 12 medical schools (9.0%) used the term ‘maternity leave.’ Semantics surrounding parental leave are important for promoting inclusivity across all family units.

### Recommendations

It may be timely for medical schools to re-evaluate and reflect upon the adequacy of their parental leave offerings and how closely they align with current medical associations’ recommendations in terms of both length and compensation. The authors recommend that policies governing parental leave for academic faculty align with the current literature and medical associations’ evidence-based suggestions, including full compensation and a continuation of benefits (i.e., health insurance coverage) to help promote the best possible family health outcomes. We echo Allan et al.’s recommendations, which state, ‘Parental leave policies should be accessible, consistent, and applicable to everyone in the workplace, including birthing and non-birthing parents, and those adopting or fostering. The leave should be of adequate duration to support the physical and mental health benefits for parents and children’ [63].

As noted in previous studies on medical school parental leave policies, the wording of these benefits is often vague and vulnerable to misinterpretation [22,23]. To promote inclusivity across all family units, it is recommended that medical schools avoid gendered language or differences in their parental leave offerings based on birthing status.

Faculty faced with ambiguous policy information and/or policies that are generally hard to locate may be vulnerable to taking less leave or compensation than they are entitled to. To avoid policy ambiguities and mischaracterizations, the authors recommend that parental leave policies stand alone, be readily accessible, include clearly defined terms and specific language, and be periodically reviewed/revised so individuals can use this information proactively when making important and private family planning decisions. For additional recommendations, see the ACOG and AMA [9,10].

### Future research directions

The provision of future state laws (e.g., paid leave laws) and/or revisions to existing parental leave policies would undoubtedly alter the accuracy of this study’s outcomes and necessitate additional follow-up studies to capture the effects of temporal changes. As such, an ongoing evaluation of medical school faculty benefits will remain important to ensure benefits offerings meet workforce needs and are in lockstep with contemporary medical associations’ recommendations for optimizing recovery of the birthing parent, child development, and parent/child bonding. Investigations concerning the effects of other factors (e.g., institutional wealth and the cost burden of parental leave) on the quality of parental leave offerings are also warranted. This study did not account for employer benefits offered to clinical faculty contracted through independent medical groups (i.e., separate corporations distinct from the medical school/university). As such, further studies investigating variations in parental leave policies between medical schools and independent medical groups may be warranted.

### Limitations

While this study represents the most comprehensive review of faculty parental leave policies across U.S. allopathic medical schools, it is not without limitations. The language of several parental leave policies was subject to different interpretations. The aim to achieve consistency in interpretation was met with challenges when policies included a variety of wording variations. For example, less commonly used policy names such as ‘Parental Workload Relief Plan,’ ‘Primary Caregiver Leave,’ and ‘Baby Bonding Time’ made it challenging to identify and categorize certain policies. Additionally, of the 156 U.S. allopathic medical schools, 22 (14.1%) had parental leave benefits behind a firewall or otherwise not available for review. We also acknowledge some employee benefits may be negotiated and instituted at the university level, and the level of policy handling may vary by institution. As such, this study evaluated parental leave policies without contextual knowledge of the level of policy handling. Therefore, we acknowledge that some medical schools may not have direct influence over their faculty benefits offerings. To the authors’ knowledge, all parental leave information was current at the time of data collection.

## Conclusions

Paid parental leave is an important benefit with many well-known and documented advantages to physical and mental health, child growth and development, and family stability. For these reasons, medical associations (e.g., AAP and AMA) have endorsed 12 weeks of fully paid parental leave after the birth, adoption, or fostering of a child. Despite this recommendation, only one-fifth (21.6%, 29/134) of parental leave policies offered to medical school faculty provided 12 weeks of birthing parent leave with full compensation. This study challenges academic medicine employers to introspectively evaluate the quality of their parental leave policies and, as needed, work toward better aligning local policies with national medical associations’ recommendations.
